# Physical activity preferences across demographic groups: a systematic review of population-based evidence and implications for public health and intervention design

**DOI:** 10.3389/fpubh.2025.1725783

**Published:** 2026-01-09

**Authors:** Carlos Martín-Martínez, Miriam García-González, Juan-José Mijarra-Murillo, José-Manuel Delfa-de-la-Morena

**Affiliations:** Department of Physiotherapy, Occupational Therapy, Rehabilitation and Physical Medicine, Universidad Rey Juan Carlos, Madrid, Spain

**Keywords:** demographic factors, exercise behavior, health promotion, physical activity adherence, population health management, sedentary behavior

## Abstract

**Background:**

Regular physical activity (PA) is a cornerstone of public health; however, participation rates remain below recommended levels worldwide. Understanding PA preferences across demographic groups can support the development of more engaging and sustainable interventions.

**Methods:**

This systematic review followed PRISMA 2020 guidelines and was prospectively registered in PROSPERO (CRD420250650308). Systematic searches were conducted in PubMed, Scopus, PsycINFO, and Web of Science (WoS) from inception to September 2025. Studies were included if they assessed PA preferences using validated instruments in general populations and excluded if they were qualitative, clinical, or lacked explicit preference assessment. Two reviewers independently extracted data on demographics, preference patterns, instruments, and study quality using a standardized template. Methodological quality was evaluated with the AXIS tool. Given heterogeneity across studies, subgroup analyses were conducted by age, gender, and socioeconomic status.

**Results:**

Twenty-two studies met inclusion criteria. Younger individuals preferred high-intensity, competitive, and social activities, whereas older adults favored low-impact, health-oriented, and independent forms of exercise. Males preferred competitive and strength-based activities, while females favored endurance and group-based options. Socioeconomic factors influenced choices, with lower-income groups more often engaging in community-based and supervised activities. Validated instruments, including the Activity Preference Assessment (APA), Decisional Preference in Exercising Test (DPEX), and Questionnaire on Physical Activity Preferences (QPAP), showed strong reliability and validity.

**Conclusion:**

Integrating preference assessment into public health strategies can enhance participation and long-term adherence to active lifestyles. Tailoring interventions to demographic-specific preferences may improve the effectiveness and equity of physical activity promotion.

**Systematic review registration:**

https://www.crd.york.ac.uk/PROSPERO/view/CRD420250650308, Identifier: CRD420250650308.

## 1 Introduction

Regular engagement in physical activity (PA) is a cornerstone of public health, associated with substantial reductions in morbidity and mortality from noncommunicable diseases such as cardiovascular disease, diabetes, and certain cancers. Despite extensive evidence on the benefits of PA, global participation rates remain below recommended levels, with only one in four adults meeting the World Health Organization (WHO) guidelines (1). Understanding the underlying determinants that influence individuals’ participation choices is therefore essential to inform more effective interventions (2).

Among these determinants, preferences for physical activity, defined as individuals’ affective and cognitive inclinations toward specific types, intensities, and contexts of exercise (3), have emerged as a key factor influencing adherence and long-term engagement (4). Evidence suggests that when PA programs align with individual preferences, participation and satisfaction increase, while dropout rates decline (5). Consequently, studying PA preferences offers a pathway to personalize public health strategies and design interventions that are both appealing and sustainable across population groups.

Previous research has explored PA preferences in specific cohorts (e.g., adolescents, older adults, or gender-based groups), yet findings remain fragmented, often constrained by heterogeneous methodologies, inconsistent instruments, and limited generalizability (6). Moreover, the reliability and validity of tools used to assess PA preferences vary considerably, hindering cross-study comparisons and the development of standardized recommendations.

To address these gaps, this systematic review aims to synthesize evidence on PA preferences across demographic groups, examining age-, gender-, and socioeconomic-related variations, while also evaluating the psychometric quality and validity of instruments used to assess these preferences. By integrating findings from diverse populations and methodologies, this work seeks to inform the design of tailored, preference-based interventions that promote long-term PA engagement and improved public health outcomes (4, 7).

## 2 Methods

### 2.1 Study design and registration

This systematic review was conducted following the Preferred Reporting Items for Systematic Reviews and Meta-Analyses (PRISMA) guidelines (8). The protocol was prospectively registered in the PROSPERO database (International Prospective Register of Systematic Reviews) under the registration number CRD420250650308.

### 2.2 Search strategy

A systematic search of electronic databases, including PubMed, Scopus, PsycINFO and Web of Science (WoS), was conducted from inception to September 1st, 2025. The search strategy incorporated a combination of keywords and Medical Subject Headings (MeSH) terms related to physical activity preferences and assessment methods. The specific search terms and Boolean operators used can be found in Supplementary File 1.

### 2.3 Eligibility criteria

Studies were included if they met the following criteria:

- *Population:* Included individuals of all age groups (children, adolescents, adults, and older adults) without restrictions based on health status.
- *Outcomes:* Assessed PA preferences using validated instruments such as preference questionnaires, choice-based experiments, or observational methods.
- *Study design:* Included observational studies, cross-sectional surveys, and intervention studies that analyzed PA preferences.
- *Publication status:* Published in peer-reviewed journals in any language.

Studies were excluded if they focused exclusively on clinical or diseased populations (for example, individuals with conditions that severely limit PA participation), if they assessed physical activity participation without explicitly measuring preference, or if they were purely qualitative studies, reviews, editorials, or conference abstracts that did not provide original data.

### 2.4 Study selection

**Figure 1:**
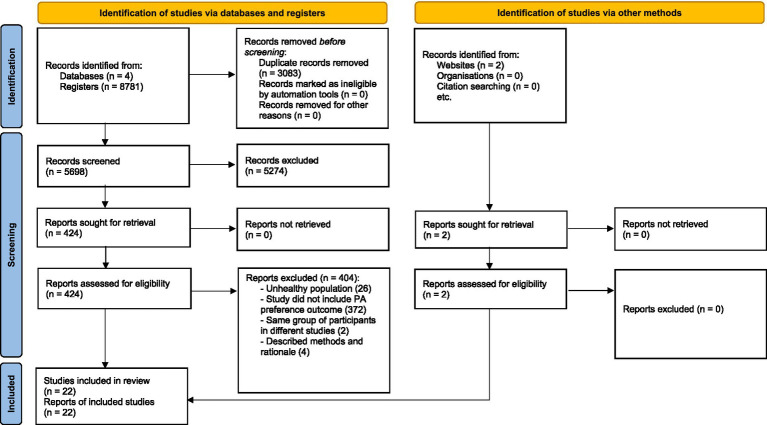
PRISMA flow diagram of literature search.

Two independent reviewers screened all retrieved records by title and abstract to determine relevance. Full-text articles were obtained when eligibility could not be confirmed from the abstract alone or when they appeared to meet inclusion criteria. Any discrepancies between reviewers were resolved through discussion and, when necessary, consultation with a third reviewer. In addition, the reference lists of all included studies were manually reviewed to identify any further relevant publications. The study selection process, including identification, screening, and inclusion, is summarized in the PRISMA flow diagram (Figure 1).

### 2.5 Data extraction and management

Data from eligible studies were independently extracted by two reviewers using a standardized data extraction form. Extracted variables included study characteristics such as author, year of publication, study design, country, and sample size; demographic details including age, sex, and socioeconomic status; and physical activity preference measures, including the type of instrument and its validation status. Key findings related to PA preferences, as well as any reported associations with PA behavior and contextual factors, were also recorded. Any inconsistencies or disagreements during the data extraction process were resolved by consensus between the reviewers to ensure accuracy and reliability.

### 2.6 Quality assessment

The methodological quality of included studies was evaluated using the AXIS tool (Appraisal tool for Cross-Sectional Studies) (9), which evaluates sampling procedures, measurement validity, response rates, and analytical transparency. The AXIS tool served not only to categorize studies as low, moderate, or high quality, but also to identify methodological patterns, e.g., inadequate reporting of non-response and insufficient justification of sample size, which were among the most common weaknesses. Each study was rated across 20 items covering design clarity, sampling, measurement, analysis, and reporting. Studies were categorized as high quality (score ≥7), moderate quality (score 5–6), or low quality (score <5). Two reviewers conducted the appraisal independently, with disagreements resolved through consensus or adjudication by a third reviewer.

### 2.7 Data synthesis

Due to extensive heterogeneity in study designs, populations, outcome measures, measurement tools and preference categories across studies, a meta-analysis was not feasible. Even when similar constructs were measured, response formats differed substantially, preventing meaningful computation of comparable effect sizes. However, we extracted directionality and consistency of associations, and Table 1 now summarizes these patterns. Future studies using harmonized preference metrics may enable quantitative pooling. A narrative synthesis was conducted, summarizing findings according to age, gender, and socioeconomic differences in PA preferences. Subgroup analyses were also performed to identify consistent demographic trends. In addition, the psychometric properties (validity and reliability) of PA preference assessment tools were analyzed and summarized.

**Table 1:** Summary of studies included in the systematic review.

Author and year	Sample demographics (*n*, mean age, sex)	Objective	Physical activity-related endpoints	Main results
Bélanger et al. (2012) (10)	*n* = 811 3.0–12.0 years	Identify PA preferences in order to provide guidance and recommendations.	Photo-pair PA preference questionnaire	Most-preferred activity type: vigorous activities (basketball, soccer and ice-skating). Younger children second choice: sedentary activities (handheld video games).
Booth et al. (1997) (23)	*n* = 2,298 18 to 39 years 40 to 59 years 60 to 78 years	Compare 3 age-groups PA preferences in order to provide guidance and recommendations.	PA preference questionnaire PA context preference questionnaire	Most-preferred activity type: walking. Youngest group preferred to exercise with a group. Oldest group preferred to exercise with a professional.
Burton et al. (2012) (22)	*n* = 7,873 42–67 years	Compare 3 age-groups PA preferences in order to provide guidance and recommendations.	PA preference questionnaire PA context preference questionnaire	Older adults preferred PA with people of same age, non-scheduled sessions, non-competitive, individual and non-vigorous. Adults with low income: PA team-based, supervised, skill-based, individual, indoor and non-vigorous. Adults with 30 + BMI: supervised PA, with people the same age and sex, team-based and scheduled sessions.
Calfas et al. (1991) (13)	*n* = 81 4.0–8.0 years 52% female	Validation of photo-pair food and exercise questionnaire (PPFEQ)	Photo-pair food and exercise questionnaire (PPFEQ)	↑ Significant correlation between test–retest questionnaire.
Cammisa et al. (2011) (29)	*n* = 49 4.0–5.0 years	Identify PA preferences in order to provide guidance and recommendations.	Focus group PA preference questionnaire	Most-preferred activity type: sedentary play (table and impersonation games).
Doyle et al. (2019) (28)	*n* = 628 20.79 ± 3.81 years 69.1% female	Identify PA preferences in order to provide guidance and recommendations	PA preference questionnaire PA context preference questionnaire	Most preferred activity types: walking (66.7%) and swimming (61.7%). Males significantly preferred competitive activities (football, fitness/weights and jogging). Females significantly preferred activity types such as walking, aerobics, cycling, squash and yoga.
Fearnbach et al. (2020) (31)	*n* = 60 8–17 years 50% female	Validation Activity Preference Assessment (APA)	Activity Preference Assessment PA levels (accelerometers) Free-play time observation Anthropometry Cardiorrespiratory fitness Socioeconomic status	↑ Significant association between Activity Preference Test and other outcomes.
Feraco et al. (2024) (24)	*n* = 2,198 41.1 ± 12.7 years 1,314 females	Compare gender PA and food preferences in order to provide guidance and recommendations.	PA preference questionnaire	Women preferred activity type: endurance and strength training. Lower percentage of women playing sports. Men preferred activity type: strength training and endurance sports.
Fromel et al. (2020) (17)	*n* = 17,032 12–25 years 22.5% female	Identify PA preferences in order to provide guidance and recommendations.	QPAP (Questionnaire on Physical Activity Preferences)	Boys most-preferred PA: ball games, running, skiing and snowboarding. Girls most-preferred PA: running, cycling, swimming, skiing and snowboarding.
Hubert et al. (2021) (30)	*n* = 251 17–18 years 18.3% 19–20 years 42.6% 21–22 years 31.5% 23 + years 7.6%	Identify PA preferences in order to provide guidance and recommendations	PA Liking Index (PAI) Healthy Eating Index PA levels and diet questionnaire Anthropometry questionnaire	↑ Significant association between sedentary activities, body size perception and dietary habits.
Jihene et al. (2015) (12)	*n* = 577 4.0–5.0 years 50% female	Intervention to improve PA and eating habits	Photo-pair PA and food preference questionnaire Parents food and PA preference questionnaire	↑ Significant changes of PA and diet were obtained in the intervention group unlike the control group.
Kudláček et al. (2015) (20)		Compare gender martial arts preferences in order to provide guidance and recommendations.	QPAP (Questionnaire on Physical Activity Preferences)	Males preferred activities: box, kick-box, karate, judo, and wrestling. Females preferred activities: karate, box, judo, kick-box and aikido
Kudlacek et al. (2020) (19)	*n* = 9,513 15–18 years 60.6% female	Identify PA preferences and levels in order to provide guidance and recommendations.	QPAP (Questionnaire on Physical Activity Preferences) IPAQ PA levels (pedometers)	Boys most-preferred PA: team sports, individual and fitness PA. Girls most-preferred PA: team sports and individual.
Kudlacek et al. (2024) (18)	*n* = 19,235 15–26 years	Identify PA preferences in order to provide guidance and recommendations.	QPAP (Questionnaire on Physical Activity Preferences)	Most-preferred activity type: individual PA (swimming, cycling and significant increase in running).
Leary et al. (2008) (14)	*n* = 17	Identify PA preferences in order to provide guidance and recommendations.	Photo-pair PA preference questionnaire Parents PA preference and levels questionnaire	↓ Non-significant correlation between parents’ perception of children PA preferences and the children actual PA preferences.
Leslie et al. (1999) (21)	*n* = 2,729 25.2 years 58% female	Identify PA preferences in order to provide guidance and recommendations.	PA preference questionnaire PA context preference questionnaire	Most-preferred activity type: racquet sports, swimming, aerobics, team sports, weight training, and walking. Males significantly preferred: weight training and team sports. Females significantly preferred: aerobics, walking, dance and yoga.
Parrish et al. (2010) (11)	*n* = 1881 4.0–9.0 years	Validation of Children’s Activity Pictures Questionnaire (CAP)	CAP Questionnaire PA levels (accelerometers) Free-play time observation	↑ Significant association between CAP Questionnaire and other outcomes.
Resaland et al. (2019) (27)	*n* = 1,026 10.2 ± 0.3 years 48% female	Analyze association between PA preferences and weight, adiposity and cardiorespiratory fitness	PA preference questionnaire Anthropometry Cardiorrespiratory fitness Socioeconomic status	↑ Significant association between sedentary activities, weight and CFR. Most-preferred activity types: soccer and slalom skiing. Most-pronounced gender differences: activities favoured by girls (dancing, gymnastics, exercising to music and jumping rope).
Sigmund et al. (2007) (16)	*n* = 111 21–24 years 22.5% female	Verify the stability of a new PA preferences survey	QPAP (Questionnaire on Physical Activity Preferences)	↑ Significant correlation between test–retest in QPAP categories.
Timme & Brand (2024) (26)	*n* = 480 28.76 ± 15.03 years 217 male	Validation Decisional Preference in Exercising Test (DPEX)	DPEX Test IPAQ Affective exercise experiences questionnaire: AFFEX Exercise e-diary: PIEL	↑ Significant association between DPEX Test and other outcomes.
Teixeira et al. (2019) (25)	*n* = 3,873 46.8 years 2046 females	Identify PA preferences in order to provide guidance and recommendations	IPAQ PA preference questionnaire Likert scale regarding daily habits	Most-preferred leisure-time activities: walking, health/fitness activities, running, group gymnastics classes, swimming/pool activities, football/futsal, and cycling. Frequently adopted commonly recommended non-sedentary activities: 15–48%
Wiseman et al. (2017) (15)	*n* = 86 3.08–5.5 years female	Validation of photo-pair food and exercise questionnaire computerized (PPFEQ)	Photo-pair food and exercise questionnaire computerized (PPFEQ)	↑ Significant correlation between test–retest questionnaire.

APA, Activity Preference Assessment; AFFEX, Affective Exercise Experiences Questionnaire; CAP, Children’s Activity Pictures Questionnaire; CFR, Cardiorespiratory Fitness; IPAQ, International Physical Activity Questionnaire; PA, Physical Activity; PAI, Physical Activity Liking Index; PIEL, Physical activity e-Diary; PPFEQ, Photo-Pair Food and Exercise Questionnaire; QPAP, Questionnaire on Physical Activity Preferences; SES, Socioeconomic Status.

## 3 Results

### 3.1 Study selection and characteristics

The flowchart of literature search is shown in Figure 1. Following full-text review, 22 studies met the inclusion criteria and were included in this review. The characteristics of the included studies are summarized in Table 1 (10–31).

These studies varied in sample sizes, ranging from small cohorts (*n* = 49) (29) to large-scale population studies (*n* = 19,235) (18), encompassing diverse age groups, genders, and socioeconomic backgrounds. The included studies employed various validated instruments to assess PA preferences, including the Activity Preference Assessment (APA) (31), the Decisional Preference in Exercising Test (DPEX) (26), the Questionnaire on Physical Activity Preferences (QPAP) (16–20), and photo-pair questionnaires such as the Photo-Pair Food and Exercise Questionnaire (PPFEQ) (13, 15).

### 3.2 Methodological quality

The quality of the included studies was overall high (median AXIS score = 7, range 6–7; Table 2). Out of the 22 studies, none of them was deemed to have poor methodological quality, six had fair quality (10, 13, 14, 16, 20, 29), and the remainder were considered to present high quality.

**Table 2:** Quality of studies included in the systematic review.

Author and year	Items																				
	1	2	3	4	5	6	7	8	9	10	11	12	13	14	15	16	17	18	19	20	Total
Bélanger et al. (2012) (10)	+	+	−	−	−	−	−	+	+	+	+	+	−	−	−	+	+	+	+	+	6
Booth et al. (1997) (23)	+	+	−	+	−	−	−	+	+	+	+	+	−	−	+	+	+	+	+	+	7
Burton et al. (2012) (22)	+	+	−	+	−	−	−	+	+	+	+	+	−	−	+	+	+	+	+	+	7
Calfas et al. (1991) (13)	+	+	−	−	−	−	−	+	+	+	+	+	−	−	−	+	+	+	+	+	6
Cammisa et al. (2011) (29)	+	+	−	−	−	−	−	+	+	+	+	+	−	−	−	+	+	+	+	+	6
Doyle et al. (2019) (28)	+	+	−	+	−	−	−	+	+	+	+	+	−	−	+	+	+	+	+	+	7
Fearnbach et al. (2020) (31)	+	+	−	+	−	−	−	+	+	+	+	+	−	−	+	+	+	+	+	+	7
Feraco et al. (2024) (24)	+	+	−	+	−	−	−	+	+	+	+	+	−	−	+	+	+	+	+	+	7
Fromel et al. (2020) (17)	+	+	−	+	−	−	−	+	+	+	+	+	−	−	+	+	+	+	+	+	7
Hubert et al. (2021) (30)	+	+	−	+	−	−	−	+	+	+	+	+	−	−	+	+	+	+	+	+	7
Jihene et al. (2015) (12)	+	+	−	+	−	−	−	+	+	+	+	+	−	−	+	+	+	+	+	+	7
Kudláček et al. (2015) (20)	+	+	−	−	−	−	−	+	+	+	+	+	−	−	−	+	+	+	+	+	6
Kudlacek et al. (2020) (19)	+	+	−	+	−	−	−	+	+	+	+	+	−	−	+	+	+	+	+	+	7
Kudlacek et al. (2024) (18)	+	+	−	+	−	−	−	+	+	+	+	+	−	−	+	+	+	+	+	+	7
Leary et al. (2008) (14)	+	+	−	−	−	−	−	+	+	+	+	+	−	−	−	+	+	+	+	+	6
Leslie et al. (1999) (21)	+	+	−	+	−	−	−	+	+	+	+	+	−	−	+	+	+	+	+	+	7
Parrish et al. (2010) (11)	+	+	−	+	−	−	−	+	+	+	+	+	−	−	+	+	+	+	+	+	7
Resaland et al. (2019) (27)	+	+	−	+	−	−	−	+	+	+	+	+	−	−	+	+	+	+	+	+	7
Sigmund et al. (2007) (16)	+	+	−	−	−	−	−	+	+	+	+	+	−	−	−	+	+	+	+	+	6
Timme & Brand (2024) (26)	+	+	−	+	−	−	−	+	+	+	+	+	−	−	+	+	+	+	+	+	7
Teixeira et al. (2019) (25)	+	+	−	+	−	−	−	+	+	+	+	+	−	−	+	+	+	+	+	+	7
Wiseman et al. (2017) (15)	+	+	−	+	−	−	−	+	+	+	+	+	−	−	+	+	+	+	+	+	7

Column numbers correspond to the following criteria on the AXIS scale: 1 – objectives of the study clear; 2 – study design appropriate; 3 – sample size justified; 4 – population clearly defined; 5 – sample included represents clearly population; 6 – selection process appropriate; 7 – non-responders considered; 8 – outcomes measured correctly; 9 – correct instruments; 10 – clear statistical analyses; 11 – results clearly described; 12 – study design appropriate; 13 – correct response rate; 14 – non-responders results described; 15 – results internally consistent; 16 – all results presented; 17 – discussion and conclusion justified; 18 – limitations discussed; 19 – conflicts of interest considered; 20 – ethical approval or consent obtained. A total score out of 10 is determined from the number of criteria that are satisfied. + Indicates the criterion was clearly satisfied; − indicates that it was not clearly satisfied.

### 3.3 Physical activity preferences by demographics

#### 3.3.1 Age-related variations

PA preferences varied considerably across age groups. Younger populations (children and adolescents) demonstrated a preference for high-intensity and social activities such as team sports, running, and cycling. In contrast, middle-aged and older adults favored lower-intensity, independent, and health-focused activities such as walking, swimming, and yoga. Studies indicated a shift from structured, competitive sports in adolescence to recreational and wellness-oriented activities in adulthood.

Children (3–12 years) preferred vigorous activities such as basketball, soccer, and ice skating (10), while some younger children also exhibited an inclination toward sedentary activities like video games (10, 29).

Adolescents (15–18 years) preferred team sports and individual fitness activities (19), with males favoring competitive sports like football and weight training, while females leaned toward group-based fitness activities like yoga and aerobics (17, 18, 28).

Among adults (18–67 years), walking emerged as the most preferred activity across all studies (22, 23, 25, 28). Other findings included: Young adults (18–39 years) preferred group-based activities (23). Middle-aged adults (40–59 years) engaged more in structured fitness activities such as gym-based exercise (25). Older adults (60 + years) demonstrated a preference for low-impact, supervised, and socially engaging activities (22, 23), including exercise programs facilitated by professionals.

#### 3.3.2 Gender differences

Consistent gender-based differences in PA preferences were observed across studies. Males preferred competitive and strength-based activities such as football, weight training, and martial arts (e.g., boxing, judo, karate, and wrestling) (19, 20, 24). Females favored endurance-based activities and group fitness, with higher participation in walking, aerobics, dance, and yoga (17, 18, 28). Across multiple studies, walking was the most universally preferred activity among all genders (21, 23, 28).

#### 3.3.3 Socioeconomic and environmental influences

Economic disparities significantly impacted PA preferences. Individuals from lower-income backgrounds showed a higher tendency to participate in community-based, supervised, and accessible activities, while those with greater financial resources had broader opportunities to engage in structured fitness programs and recreational sports. Lower-income adults preferred team-based, supervised, skill-based, and indoor activities (22). Individuals with BMI > 30 preferred structured, supervised PA sessions with peers of the same age and sex (22). Public and accessible options such as park-based exercise programs and community sports leagues were more popular among lower socioeconomic groups (22, 23).

### 3.4 Validity and reliability of PA preference assessments

Several studies validated PA preference assessment tools, demonstrating strong associations between preference measures and actual PA behaviors. The APA and DPEX showed strong correlations with PA levels measured via accelerometers and self-reported questionnaires (26, 31). The APA (31) was significantly correlated with free-play time, cardiorespiratory fitness, and socioeconomic status. The DPEX (26) showed strong reliability when compared to IPAQ (International Physical Activity Questionnaire) and affective exercise experiences. The QPAP (16–20) was consistently used to assess PA trends in adolescents and adults. The PPFEQ (13, 15) was validated in children, showing significant test–retest correlations.

### 3.5 Narrative summary of effect patterns across studies

Across the included studies, several consistent demographic-related patterns emerged in PA preferences. Age showed the clearest directional trend: children and adolescents consistently preferred vigorous, social, and competitive activities, while adults and older adults favored low-intensity, health-oriented, and independent forms of PA (10, 17, 18). Gender differences were also highly consistent, with males preferring competitive and strength-based activities (19, 20, 24), and females demonstrating a stronger inclination toward endurance, group-based, and rhythm-oriented activities (17, 18, 28). Socioeconomic status revealed a pattern in which individuals with lower income or educational attainment preferred community-based, supervised, and accessible activities (22), whereas those with higher socioeconomic status engaged more frequently in structured fitness programs and recreational sports (22, 23). These directional trends were observed across different study designs and instruments, supporting the robustness of the findings despite methodological heterogeneity. A structured summary of these associations is presented in Table 3.

**Table 3:** Summary of direction and consistency of associations between demographic factors and physical activity preferences across studies.

Demographic factor	Direction of association	Consistency across studies	Example findings	Representative studies
Age	Younger → vigorous, competitive, social. Older adults → low-intensity, health-oriented, independent.	High	Children prefer team sports, running, cycling. Older adults prefer walking, swimming, supervised PA	(10, 17, 18, 22, 23, 25, 27, 28)
Gender	Males → competitive, strength-based. Females → endurance, group-based, rhythmic.	High	Men prefer football, weight training. Women prefer walking, aerobics, dance, yoga	(17–21, 24, 28)
Socioeconomic Status	Lower SES → supervised, accessible, community-based activities. Higher SES → structured programs, recreational sports.	Moderate–High	Lower SES: supervised team-based PA. Higher SES: gym-based fitness, individualized programs.	(22, 23, 25)
BMI/weight Status	Higher BMI → preference for supervised, age−/sex-matched, low-intensity sessions	Moderate	Individuals with BMI > 30 show preference for structured and supervised PA.	(22, 27)
Urban vs. Rural context	Urban → structured fitness and individual activities. Rural → outdoor and socially embedded activities.	Moderate	Rural youth show higher preference for locally accessible sports.	(27, 28)
Cultural context/country	Western countries → broader variety of choices; Limited evidence from LMICs restricts pattern estimation	Low (due to sparse data)	Preferences influenced by access and cultural norms.	Limited across all studies

BMI, Body Max Index; LMICs, Low- and Middle-Income Countries; PA, Physical Activity; SES, Socioeconomic Status.

### 3.6 Summary of main findings

Walking emerged as the most universally preferred activity across all age groups and genders (22, 23, 25, 28).

Younger individuals favored high-intensity, competitive, and social activities, while older adults preferred low-impact, structured, and professionally guided exercises (10, 17, 18).

Gender differences were consistently observed. Males preferred competitive, strength-based sports (19, 20, 24), while females gravitated toward endurance and group-based activities (17, 18, 28).

Socioeconomic factors significantly influenced PA preferences. Lower-income populations opted for community-based and supervised activities (22), whereas higher-income individuals engaged in structured fitness programs and recreational sports (22, 23).

Validated PA preference assessment tools (APA, DPEX, QPAP, and PPFEQ) demonstrated strong reliability and accuracy, reinforcing their utility in designing targeted interventions (15, 18–20, 26, 31). These findings underscore the importance of tailoring PA interventions based on demographic factors to enhance engagement and long-term adherence.

## 4 Discussion

### 4.1 Summary of main findings

This systematic review provides a comprehensive synthesis of evidence regarding PA preferences across demographic groups. By consolidating findings from 22 studies of diverse populations, the review highlights consistent age-, gender-, and socioeconomic-related trends that shape engagement in physical activity. The results emphasize that preference is not a trivial factor but a central determinant of participation, influencing both initiation and long-term adherence to active behaviors (4, 5).

### 4.2 Interpretation in the context of previous evidence

A clear age-related gradient was observed: younger individuals tended to prefer vigorous, competitive, and socially interactive activities, while older adults favored lower-intensity, self-paced, and health-oriented forms of PA. This aligns with developmental and motivational theories suggesting that social reinforcement and competition drive participation in youth, whereas autonomy, functionality, and health preservation become more salient motivators in adulthood and later life (32). Importantly, these patterns also correspond to key constructs of Self-Determination Theory (SDT), whereby younger individuals’ preferences reflect needs for competence and relatedness, while the shift toward self-paced and health-oriented activities in older adults illustrates a stronger orientation toward autonomous motivation. The transition from team-based to wellness-based preferences therefore supports both SDT principles and a life-course approach to promoting PA engagement (33).

Gender differences were also consistent across studies. Males showed a stronger preference for competitive and strength-based activities, reflecting traditional gender norms and motivational differences such as mastery orientation and performance goals. Conversely, females preferred group-based and endurance-oriented activities that often emphasize social connection, rhythm, and self-expression (34). These distinctions are consistent with prior evidence showing that women tend to value social interaction and enjoyment, while men prioritize performance and competition in PA contexts (25). This gender-specific pattern further aligns with motivational frameworks suggesting that competence-driven activities may resonate more strongly among males, whereas relatedness-driven contexts may be more appealing to females.

Socioeconomic and environmental factors also emerged as significant influences on PA preferences. Lower-income groups favored community-based and accessible forms of activity, highlighting the importance of environmental and financial accessibility. These findings support ecological models of health behavior, which recognize that opportunity structures (such as public spaces and affordable programs) mediate the translation of preference into behavior (35). Designing interventions that account for these contextual barriers is therefore critical to promoting equity in PA participation (36). Integrating socio-ecological perspectives with motivational theory suggests that preference is shaped not only by individual inclinations but also by structural conditions that facilitate or constrain behavioral options.

### 4.3 Methodological considerations and quality of evidence

A novel contribution of this review is its synthesis of psychometric evidence for PA preference instruments. The APA (31), DPEX (26), QPAP (16–20), and PPFEQ (13, 15) all demonstrated adequate validity and reliability across populations. However, few studies examined cross-cultural equivalence or sensitivity to change, limiting their broader applicability (6). Future research should focus on validating these instruments in diverse cultural and socioeconomic settings to strengthen their use in global public health surveillance.

Among reviewed tools, the APA (31) and DPEX (26) are best suited for research contexts due to their detailed psychometric validation and ability to infer latent preference structures. By contrast, the QPAP (16–20) and PPFEQ (13, 15) are more practical for surveillance and large-scale monitoring because of their brief administration time and straightforward scoring.

### 4.4 Limitations and strengths

This review’s strengths include adherence to PRISMA 2020 standards (8), prospective registration in PROSPERO, and a rigorous quality assessment using the AXIS tool. The inclusion of studies across all age groups and socioeconomic strata enhances generalizability, and the synthesis of psychometric evidence adds methodological depth rarely addressed in prior reviews. A major strength of this review is the comprehensive inclusion of validated instruments across all demographic groups and the use of a standardized appraisal tool (AXIS) (9).

However, several limitations should be noted. First, heterogeneity across study designs and measurement tools precluded meta-analytic synthesis. Second, most included studies were cross-sectional, limiting causal interpretation of the relationship between preference and PA behavior. Third, although only validated instruments were included, variability in their validation criteria may have influenced comparative analyses. This decision to include only validated instruments aimed to ensure psychometric rigor and comparability across studies. However, we acknowledge that this criterion may have excluded emerging or culturally adapted tools commonly used in non-Western settings. While this approach enhances internal validity, it may inadvertently limit cultural diversity in the included evidence. Future reviews may consider a two-tiered approach in which validated and non-validated tools are analyzed separately. Finally, although this review includes diverse population groups, most studies were conducted in high-income countries such as Australia, the United States, and European nations. This overrepresentation limits the external validity of our findings, particularly for low- and middle-income countries, where sociocultural norms, environmental constraints, and access to facilities may substantially shape activity preferences. Future research should prioritize culturally grounded methodologies and the validation of preference instruments within low- and middle-income countries contexts to ensure globally relevant evidence.

### 4.5 Implications for practice, policy, and future research

The findings underscore the potential of preference-based approaches in designing PA promotion strategies. Incorporating individual preferences into intervention design can enhance engagement, motivation, and adherence (critical elements for sustained behavioral change) (37, 38). Public health practitioners should therefore use validated tools to identify population-specific activity preferences and tailor interventions accordingly (2). For instance, promoting social, team-based activities among adolescents or offering supervised, low-impact options for older adults may increase participation rates and reduce inactivity disparities.

From a policy perspective, integrating preference assessment into national health surveys could provide actionable insights for targeted program design. Moreover, leveraging preference data in digital health tools or exercise prescription platforms may support personalized PA recommendations aligned with user motivations and contexts (39).

Future studies should adopt longitudinal and intervention designs to explore how preferences evolve over time and how alignment between preference and PA type affects long-term adherence, in line with updated global guidelines emphasizing tailored strategies (1). Review expansion into low- and middle-income countries is particularly needed given that physical activity preferences may be shaped by different cultural, environmental, and economic constraints. Incorporating research from low- and middle-income countries would not only broaden the diversity of preference profiles identified but also support the adaptation of preference-based interventions to resource-limited settings. Additionally, developing standardized, cross-culturally validated preference tools will facilitate more consistent assessment and enhance the integration of preference-based strategies into health promotion programs worldwide.

Based on the synthesis, we propose preference-based strategies for key demographic groups:

- *Adolescents:* prioritize team-based and socially rich environments; integrate competition and digital gamification.
- *Young adults:* offer flexible group classes and high-intensity options.
- *Middle-aged adults:* emphasize structured fitness routines and time-efficient formats.
- *Older adults:* provide supervised, low-impact, socially supportive programs.
- *Low-income populations:* expand access to free or low-cost community programs, walking groups, and park-based options.

## 5 Conclusion

This systematic review demonstrates that preferences for physical activity vary consistently across demographic groups and are shaped by age, gender, and socioeconomic status. Younger individuals favor vigorous and social forms of activity, whereas older adults prefer low-impact, health-oriented, and supervised exercise. Gender differences reveal that males tend to prefer competitive and strength-based activities, while females are more inclined toward endurance and group-based formats. Socioeconomic disparities influence both access and choice, underscoring the importance of environmental and contextual factors.

Validated tools such as the APA, DPEX, QPAP, and PPFEQ showed strong psychometric properties, supporting their use in research and public health practice. Integrating preference assessment into intervention design can enhance participation, motivation, and long-term adherence to active lifestyles. These findings highlight the value of preference-based approaches in health promotion strategies and support the development of tailored programs that address individual motivations and contextual realities.

## Data Availability

The original contributions presented in the study are included in the article/Supplementary material, further inquiries can be directed to the corresponding author.
