# Supplementary material for: Physical activity preferences across demographic groups: a systematic review of population-based evidence and implications for public health and intervention design
Source: Front Public Health. 2026 Jan 9;13:1725783. doi: 10.3389/fpubh.2025.1725783 (PMC12827602; doi:10.3389/fpubh.2025.1725783)
Supplement: Supplementary file 1 [file Table_1.docx]

Supplementary file 1. Search strategy

**PubMed**

Search code: ("physical activ"[Title/Abstract] OR "physical exercise"[Title/Abstract] OR sport[Title/Abstract] OR "physical education"[Title/Abstract] OR "physical training"[Title/Abstract]) AND ("data collection"[Title/Abstract] OR "technique measurement"[Title/Abstract] OR assessment[Title/Abstract] OR evaluation[Title/Abstract] OR questionnaire[Title/Abstract]) AND (preference[Title/Abstract] OR "liking"[Title/Abstract] OR "choice"[Title/Abstract])

**Scopus**

Search code: TITLE-ABS(("physical activ" OR "physical exercise" OR sport OR "physical education" OR "physical training") AND ("data collection" OR "technique measurement" OR assessment OR evaluation OR questionnaire) AND (preference OR "liking" OR "choice"))

**PsycINFO**

Search code: ti("physical activ" OR "physical exercise" OR sport OR "physical education" OR "physical training") AND ti("data collection" OR "technique measurement" OR assessment OR evaluation OR questionnaire) AND ti(preference OR "liking" OR "choice")

Search code: ab("physical activ" OR "physical exercise" OR sport OR "physical education" OR "physical training") AND ab("data collection" OR "technique measurement" OR assessment OR evaluation OR questionnaire) AND ab(preference OR "liking" OR "choice")

**Web of Science (WoS)**

Search code: ti=("physical activ" OR "physical exercise" OR sport OR "physical education" OR "physical training") AND ti=("data collection" OR "technique measurement" OR assessment OR evaluation OR questionnaire) AND ti=(preference OR "liking" OR "choice")

Search code: ab=("physical activ" OR "physical exercise" OR sport OR "physical education" OR "physical training") AND ab=("data collection" OR "technique measurement" OR assessment OR evaluation OR questionnaire) AND ab=(preference OR "liking" OR "choice")
